# Effects of Low-Frequency Solid-State Microwave Cooking on the Quality Properties of Beef Meat

**DOI:** 10.3390/foods15020214

**Published:** 2026-01-08

**Authors:** Gönül Çavuşoğlu Kaplan, Ebru Fıratlıgil

**Affiliations:** Food Engineering, Faculty of Chemical and Metallurgical Engineering, Istanbul Technical University, Ayazağa, Istanbul 34469, Turkey; ebruf@itu.edu.tr

**Keywords:** solid-state microwave cooking, microwave cooking, beef meat

## Abstract

Solid-state microwave technology has emerged as an alternative to conventional magnetron-based microwave systems due to its precise frequency control and potential to improve heating uniformity. The objective of this study was to evaluate the effects of solid-state microwave cooking at 912–913 MHz on the quality characteristics of beef steak and minced beef in comparison with conventional oven cooking and traditional microwave cooking (2450 MHz). Meat samples were cooked to an internal temperature of 75 °C, and cooking time, weight loss, moisture content, lipid oxidation (TBARS), total soluble protein (TSP), color attributes, and texture properties were evaluated. Solid-state microwave cooking resulted in shorter cooking times compared to conventional oven cooking. However, it caused significantly higher cooking loss in beef steak (48.1%) compared to conventional (34.8%) and microwave cooking (42.4%) (*p* ≤ 0.05). In minced beef, solid-state microwave cooking led to significantly higher TBARS values (1.56 mg MDA/kg) than conventional cooking (1.07 mg MDA/kg) (*p* ≤ 0.05), indicating increased lipid oxidation. No significant differences were observed among cooking methods for total soluble protein content and several texture parameters (*p* > 0.05). Solid-state microwave cooking produced improved color development compared to traditional microwave cooking. Overall, solid-state microwave cooking shows potential advantages in processing time and color formation; however, increased cooking loss and lipid oxidation indicate that optimization of processing conditions is necessary to limit undesirable quality changes.

## 1. Introduction

In recent years, the consumption of fast and convenient foods has increased, largely driven by urban lifestyles, time constraints, and changing eating habits. Social factors such as limited time for home cooking, increased reliance on ready-to-eat meals, and the growing demand for rapid food preparation have contributed to this trend. With increasing consumer demand for faster and more energy-efficient cooking, understanding how cooking methods affect meat quality has become increasingly important. The cooking process of meat induces significant physical and chemical changes, including protein denaturation, lipid oxidation, and cooking loss, all of which directly influence product quality [1]. The cooking method plays a key role in determining the extent of these transformations. While traditional methods such as oven-baking and grilling are commonly preferred for the preparation of meat products, particularly beef cuts and minced meat, microwave cooking is also widely used due to its advantages in terms of speed and energy efficiency [1,2]. Previous studies have shown that different cooking methods can markedly influence sensory-related quality attributes, including color, texture, and moisture retention, through their effects on heat transfer, protein denaturation, and water loss. In particular, microwave cooking has been associated with notable changes in meat color and appearance, which are critical factors influencing sensory quality [3].

Recently, solid-state microwave technology, a magnetron-free system using semiconductor amplifiers for precise frequency control, has gained prominence as an innovative alternative to traditional microwave technology, which typically relies on magnetron-based systems. Unlike conventional magnetrons, solid-state systems allow frequency tuning, enabling more uniform heating and improved food quality. This ability to operate at specific frequency ranges tailored to different food types offers distinct advantages in optimizing cooking performance, as frequency selection plays a crucial role in heat transfer and final product quality [4,5]. Although solid-state technology can be implemented across various ISM bands, including 2450 MHz, operation within the 915 MHz band exploits the physical advantages associated with lower frequencies. Compared to 2450 MHz, the 915 MHz range provides greater penetration depth, enabling more effective volumetric heating in food systems [5,6]. Previous studies have demonstrated the potential of solid-state microwave technology to improve cooking performance and quality attributes in plant-based foods, reporting reduced weight loss and enhanced retention of bioactive compounds compared with conventional microwave and oven cooking [6]. However, despite growing interest in solid-state microwave applications, their effects on meat systems remain largely unexplored. In particular, limited information is available regarding the influence of solid-state microwave heating on key meat quality parameters, including protein denaturation, lipid oxidation, moisture retention, color stability, and textural properties, which are critical determinants of consumer acceptance and product quality.

This study aims to evaluate the effects of solid-state microwave cooking at 912 and 913 MHz in comparison with traditional microwave cooking (2450 MHz) and conventional oven cooking on beef steak and minced beef by assessing key meat quality parameters, including weight loss, moisture content, total soluble protein content, color attributes, and texture properties. It was hypothesized that solid-state microwave cooking at lower frequencies (912–913 MHz) would result in differences in cooking behavior and selected meat quality attributes compared with conventional microwave and oven cooking methods.

## 2. Materials and Methods

### 2.1. Materials

Beef striploin (*longissimus thoracis et lumborum*) was used for steak samples, and commercially ground beef (minced beef) was used for minced beef samples. Both sample types were sourced from a local market in Istanbul, Turkey and transported to the laboratory under refrigerated conditions (≤4 °C). Samples were kept in their original retail packaging and additionally placed in sealed polyethylene bags during transport to prevent moisture loss and contamination. These beef striploin steaks and minced beef samples were selected to minimize compositional and structural variability between samples (fat content, visible connective tissue, etc.) thereby improving repeatability. Only fresh samples exhibiting normal color, texture, and water-holding properties were selected. Samples showing excessive drip loss or visible indicators of stress-related defects were excluded to minimize variability associated with pre-slaughter conditions.

Upon arrival at the laboratory, the samples were wrapped in polyethylene film and stored at 4 °C for no longer than 24 h before cooking and analysis. Beef striploin steaks were cut perpendicular to the muscle fiber into discs with a diameter of 100 ± 5 mm and a thickness of 10 ± 0.5 mm, while ground beef samples were manually homogenized and shaped into 100 mm × 100 mm × 10 mm squares using a stainless-steel mold. For each cooking method, three independent samples were prepared for both beef steak and minced beef.

All samples were cooked until reaching an internal core temperature of 75 °C, which was monitored using a calibrated type-K thermocouple probe (Testo SE & Co. KGaA, Titisee-Neustadt, Germany) inserted into the geometric center of the samples. Cooking conditions for each method are summarized in Table 1.

### 2.2. Solid-State Microwave System

**Figure 1:**
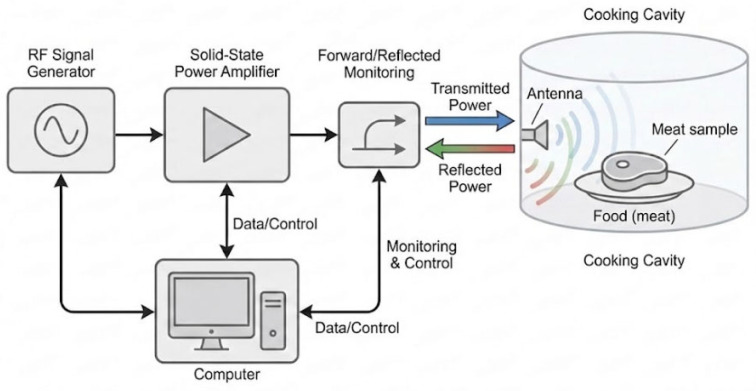
Schematic diagram of the solid-state microwave cooking system.

The experimental setup was adapted from a previous study, which was designed to monitor and calculate the microwave energy transfer efficiency in real-time [6]. The system consisted of a vector signal generator (Keysight N5172B, EXG, 9 kHz–6 GHz, Santa Rosa, CA, USA), a solid-state RF power amplifier (IFI SMV350, 500 MHz–1 GHz, Reinach, Switzerland), a high-quality coaxial cable, and a centrally mounted monopole antenna positioned within a custom-designed cooking cavity. A computer algorithm controlled the power flow and dynamically monitored transmitted and reflected signals throughout the heating process. A schematic representation of the solid-state microwave cooking system is shown in Figure 1.

For each sample, the optimum frequency was determined within the 902–928 MHz band by scanning for the frequency that produced the highest forward power with the least reflection. Once selected, the microwave signal was amplified to 350 W and directed into the cavity via the antenna. The reflected signal was captured and analyzed to determine real-time efficiency. Microwave heating efficiency was calculated using the following formula:

$$\text{Efficiency rate}=\frac{\text{Power transmitted into the cavity}\left(W\right)-\text{Reflected power from the cavity}\left(W\right)}{\text{Power transmitted into the cavity}\left(W\right)}$$

### 2.3. Weight Loss

The weight loss of the meats was determined using the following formula at the end of the cooking, as commonly applied in meat cooking studies [6]:

$$\left(\%\right)\text{Weight loss}=\frac{\text{Weight of the raw sample}\left(g\right)-\text{Weight of the cooked sample}\left(g\right)}{\text{Weight of the raw sample}\left(g\right)}\times 100$$

### 2.4. Moisture, Fat and Protein

Moisture, fat, and protein contents of the meat samples were determined according to AOAC official methods. Moisture content was measured using a halogen moisture analyzer (HB43, Mettler Toledo Inc., Columbus, OH, USA). Fat content was determined by Soxhlet extraction using petroleum ether, while protein content was analyzed by the Kjeldahl method, with nitrogen content converted to protein using a factor of 6.25. All analyses were performed in triplicate following standard AOAC procedures [7].

### 2.5. Thiobarbituric Acid

Thiobarbituric acid reactive substances (TBARS) were determined based on a previous study with slight modifications [8]. A 15 g meat sample was homogenized with 30 mL of 7.5% trichloroacetic acid (TCA) using a laboratory blender (Waring Commercial, Stamford, CT, USA) at 18,000 rpm for 1 min, filtered through Whatman No. 1 filter paper (Merk, Darmstadt, Germany) and centrifuged at 5000 rpm at 4 °C. Then, 5 mL of the supernatant was mixed with 5 mL of 20 mM thiobarbituric acid (TBA), incubated in a water bath at 85 °C for 45 min, and cooled to room temperature. Absorbance was measured at 532 nm using a UV–Vis spectrophotometer (PerkinElmer, Shelton, CT, USA) in 1 cm path-length cuvettes, with a reagent blank. A standard calibration curve was prepared using 1,1,3,3-tetramethoxypropane (TMP) in 7.5% TCA, and the method showed good linearity (R^2^ > 0.99). TBARS values were expressed as mg malondialdehyde (MDA) per kg of meat.

### 2.6. Total Soluble Protein

Total soluble protein was assessed using a modified version of the Bradford method [9]. A 2 g meat sample was homogenized with 20 mL of extraction buffer and centrifuged at 14,000 rpm for 15 min. Then, 100 μL of the supernatant was mixed with 5 mL of Bradford reagent. After incubation for 5 min at room temperature, absorbance was measured at 595 nm in a 1 cm path-length cuvette against a reagent blank using a UV–Vis spectrophotometer (PerkinElmer, Shelton, CT, USA). A standard calibration curve was prepared using bovine serum albumin (BSA) in the range of 0–1.0 mg/mL, and the calibration curve had an R^2^ value greater than 0.99. Results were expressed as mg soluble protein per g dry matter.

### 2.7. Color Parameters

The color parameters (CIE Lab* color space) of meat samples were determined using a spectrophotometer (CM-5, Konica Minolta, Tokyo, Japan) under standard measurement conditions. The L*, a*, b*, Chroma (C*), and Hue angle (h°) values were directly obtained from the instrument. Measurements were taken at three evenly distributed locations on the sample surface, avoiding visible fat, connective tissue, and surface irregularities, to ensure representative color measurements.

### 2.8. Texture Measurements

Textural measurements were performed using a texture analyzer (TA.XTplus100C, Stable Micro System, Godalming, England) equipped with a 5 kg load cell and a Warner Bratzler Blade Set with a “V” slot blade operating at a speed of 5 mm/s. The instrument’s software (Texture Exponent, Version 6.1.15, Stable Micro System, Surrey, England) was used to calculate the firmness and toughness [10,11]. Meat samples were cut into uniform strips, and the blade was aligned perpendicular to the direction of muscle fibers. Three replicate measurements were performed for each cooking condition.

### 2.9. Statistical Analysis

Data were collected from three independent steak or mince preparations for each cooking method. For each replicate, all analytical measurements were conducted in triplicate, and results were expressed as mean ± standard deviation. The normal distribution of the data was assessed using the Kolmogorov–Smirnov test. One-way analysis of variance (ANOVA) was performed using Minitab 16 Statistical Software for Windows^®^ (Minitab Inc., State Collage, PA, USA), and Tukey’s multiple comparison test was used to determine significant differences among treatment groups. Statistical significance was considered at *p* ≤ 0.05.

## 3. Results and Discussion

### 3.1. Cooking Frequency and Time

**Figure 2:**
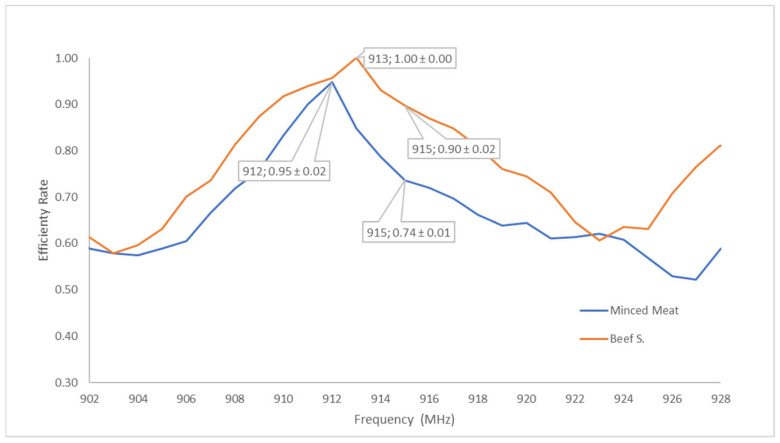
Efficiency ratio variation by frequency for beef steak and minced beef.

Prior to the experimental studies, the effective frequency values for solid-state microwave cooking on beef steak and minced beef were determined. Before cooking, the transmitted and reflected power levels in the 902–928 MHz frequency range were monitored (Figure 2).

As expected, the efficiency values of both types of samples change with frequency. The energy absorbed changes as the form of the food, and its fat and water content vary. Although the industry standard center frequency is 915 MHz, as shown in the graph, the highest efficiency (1.00) is achieved at 913 MHz for steak, while the highest efficiency ratio (0.95) for minced beef is obtained at 912 MHz. Therefore, solid-state microwave cooking tests for both types of food have been conducted at these determined frequencies.

Table 2 shows the cooking times for traditional oven cooking, microwave cooking, and solid-state microwave cooking. It is expected that beef steak will have longer cooking times compared to minced beef. In the studies conducted, it has been indicated that during microwave cooking, the cooking time decreases as the fat level of the meat increases [12]. On the other hand, these results show that the conventional microwave method significantly reduces cooking times for meat samples compared to other methods. Although solid-state microwave cooking takes longer than conventional microwave cooking, it yields faster results compared to traditional cooking. These results are similar to the findings obtained with vegetable samples [6]. There could be several potential reasons why cooking times at 912 and 913 MHz are longer than at 2450 MHz. This situation is thought to be due to the effects of energy transmission at low frequency on penetration depth and energy density.

### 3.2. Weight Loss (%), Moisture (%), Fat (%), and Protein (%)

The impact of cooking method on weight loss, moisture content, fat, and protein levels in beef steak and minced beef is shown in Table 3.

Overall, weight loss significantly varied depending on the cooking method used (*p* ≤ 0.05). In beef steak, solid-state microwave cooking resulted in significantly higher weight loss (48.1%) compared to conventional cooking (34.8%) (*p* ≤ 0.05), while microwave cooking (42.4%) showed intermediate values that were not significantly different from either treatment. Similarly, in minced beef, weight loss values increased from conventional cooking (24.5%) to microwave (26.7%) and solid-state microwave cooking (28.9%), with solid-state microwave cooking showing significantly higher weight loss compared to conventional cooking (*p* ≤ 0.05). This trend is consistent with previous findings showing that microwave-based cooking promotes more rapid heat transfer, leading to increased moisture evaporation and fat melting [13,14]. As expected, moisture content decreased as weight loss increased. In steak samples, solid-state microwave cooking caused the greatest moisture loss (*p* ≤ 0.05). The reduced moisture content in conventional cooking may be related to the surface sealing effect, as previously noted [15,16]. In minced beef, moisture differences between methods were not statistically significant (*p* > 0.05), but slightly higher moisture retention in conventional and microwave methods may relate to loss of non-water components during cooking.

Fat content in raw minced beef was considerably higher (14.2%) than in steak (1.4%). After cooking, the fat content in minced beef decreased to 11.5% (conventional), 10.3% (microwave), and 11.6% (solid-state microwave), primarily due to melted fat separating from the meat matrix during heating. Although numerical differences were observed among cooking methods, these changes were not statistically significant (*p* > 0.05), likely due to within-group variability and the higher initial fat content of minced beef [17].

Protein content showed a decrease after conventional cooking but increased slightly in microwave and solid-state methods. However, differences were not statistically significant (*p* > 0.05). The increased protein concentration in microwave conditions may be due to more rapid water loss, concentrating residual components. Electromagnetic effects may also alter protein structure and tissue integrity, enhancing loss of bound water and soluble molecules [18,19].

### 3.3. Thiobarbituric Acid Reactive Substances (TBARS) and Total Soluble Protein (TSP)

Lipid oxidation and protein denaturation are key quality indicators influenced by thermal processing in meat. TBARS and TSP values, as shown in Table 4, were used to assess these parameters in both beef steak and minced beef.

TBARS values in beef steak were statistically similar between raw and cooked samples (0.52-0.61 mg MDA/kg), indicating that cooking method did not significantly affect lipid oxidation in steak. In minced beef, which has a higher fat content and greater surface area, TBARS values increased from 0.76 mg MDA/kg in raw samples to 1.07 mg MDA/kg, 1.44 mg MDA/kg, and 1.56 mg MDA/kg after conventional, microwave, and solid-state microwave cooking, respectively, with significant differences observed among cooking methods (*p* ≤ 0.05). This corresponds to an approximate 105% increase for solid-state microwave cooking in minced beef, compared to about 17% in steak. The higher TBARS values observed in minced samples can be attributed to their greater susceptibility to oxidation due to increased fat content and disrupted structure. Similar increases in lipid oxidation following microwave cooking have been reported in red meat systems, where rapid heating and electromagnetic effects were associated with enhanced TBARS formation [17]. Previous studies in other meat products have also reported enhanced lipid oxidation following microwave processing; for instance, microwave-cooked chicken nuggets exhibited significantly higher TBARS levels, possibly due to electromagnetic interactions with lipids that enhance dipole rotation and molecular vibration, thereby promoting free radical formation and accelerating the oxidation of polyunsaturated fatty acids, even at shorter cooking times [20].

However, some studies suggest that while TBARS values increase after cooking, the difference among methods may not always be statistically significant [21]. Importantly, all TBARS values in this study remained below the 2 mg MDA/kg level reported as a limiting threshold for consumer acceptability of oxidation-altered flavour in beef [22].

TSP values, indicative of heat-induced protein denaturation, decreased substantially after cooking. In beef steak, the raw TSP content of 140.01 mg/g dry matter decreased to 14.22 mg/g (conventional), 6.11 mg/g (microwave), and 9.01 mg/g (solid-state microwave). Similarly, in minced beef, TSP declined from 92.02 mg/g to 7.56 mg/g, 12.32 mg/g, and 13.12 mg/g, respectively. These changes were statistically significant when compared to raw samples (*p* ≤ 0.05), whereas no significant differences were observed among the different cooking methods (*p* > 0.05). The loss of soluble protein can be attributed to heat-induced denaturation and aggregation of sarcoplasmic and myofibrillar proteins, membrane disruption, and contraction of connective tissues [23,24].

Interestingly, microwave and solid-state microwave treatments resulted in slightly higher residual TSP levels in minced beef compared to conventional cooking. This may reflect differences in heat distribution and exposure duration. These results align with previous findings showing substantial decreases in protein solubility following thermal treatments, though not always significantly different across cooking methods [25,26].

Furthermore, protein solubility is closely related to water-holding capacity. Denatured proteins cause meat fibers to shrink and create inter-fiber spaces, affecting both moisture retention and texture. The extent of shrinkage also varies depending on the type of muscle and thermal gradient [27], providing a possible explanation for the observed parallel between TSP and weight/moisture loss trends in both meat types.

### 3.4. Color

The color of meat is a critical quality attribute, primarily influenced by myoglobin content and its thermal denaturation. During cooking, myoglobin—responsible for the red color of raw meat—denatures due to heat exposure, altering the pigment structure and resulting in visible color changes. Additionally, Maillard reactions occurring at high surface temperatures play a significant role in crust color formation during cooking [3]. The effects of various cooking methods on color parameters (L*, a*, b*, C, and Hue) in beef steak and minced beef are presented in Table 5.

In beef steak, the L* value (lightness) increased from 40.80 in raw samples to 47.61 and 47.37 after conventional and microwave cooking, respectively, indicating enhanced brightness due to water loss and surface denaturation. Solid-state microwave cooking resulted in a slightly lower L* value (44.12), likely due to deeper heat penetration at 913 MHz, leading to less surface dehydration. A similar trend was observed in the a* value (redness), which decreased in all treatments due to myoglobin denaturation—from 20.25 in raw steak to 10.01 (conventional), 7.26 (microwave), and 8.47 (solid-state microwave). Microwave treatment caused the greatest reduction, while solid-state microwave preserved redness slightly better than conventional methods. In contrast, b* values (yellowness) showed no significant changes, ranging from 17.43 to 18.14 across all conditions.

These observations align with previous findings in lamb meat, where L* values increased while a* values decreased during cooking, with no major differences between microwave and oven heating [14].

In minced beef, significant differences in color parameters were observed among cooking methods (*p* ≤ 0.05). The initial L* value (48.38) decreased after cooking, with solid-state microwave samples showing the lowest lightness value (37.00), followed by conventional (38.31) and microwave cooking (42.39). The significant reduction in L* indicates darker surface development, likely associated with enhanced Maillard reactions and higher surface temperatures in minced beef due to its higher fat content [12]. Redness (a*) also decreased significantly after cooking (*p* ≤ 0.05), with microwave cooking resulting in the highest a* value (10.58), followed by conventional (8.48) and solid-state microwave cooking (8.37). Yellowness (b*) values increased significantly in microwave and solid-state microwave treatments compared to conventional cooking (*p* ≤ 0.05), suggesting more pronounced pigment transformation and Maillard compound formation under microwave-based heating conditions.

The increase in L* values, especially in steak samples, may be linked to protein denaturation and microstructural changes such as muscle fiber contraction, reduced water-holding capacity, and sarcoplasmic membrane damage, all of which enhance light scattering [28]. However, at higher surface temperatures, Maillard browning can reduce lightness. The lower L* values in solid-state microwave samples may be due to slower surface heating at 913 MHz, delaying crust formation and resulting in more uniform internal heating.

Chroma (C) and Hue angle offer further insight into color saturation and tone. In steak, the C value decreased from 26.71 (raw) to 20.34 (conventional), 19.57 (microwave), and 19.76 (solid-state microwave), with conventional cooking causing the greatest loss of color saturation. Hue angle increased significantly, indicating reduced redness: from 40.71 (raw) to 60.58, 68.11, and 64.70 in conventional, microwave, and solid-state microwave treatments, respectively. These changes parallel the observed a* value trends.

In minced beef, C values followed a similar pattern, dropping from 20.55 (raw) to 15.80 (conventional), 19.82 (microwave), and 17.79 (solid-state microwave). Again, lower chroma is consistent with greater protein denaturation and moisture loss. The Hue angle increased across all cooking methods—from 48.81 (raw) to 57.63 (conventional), 56.43 (microwave), and 62.61 (solid-state microwave)—reflecting the shift from red to yellow hues due to pigment degradation and Maillard products.

Overall, the differences in color parameters between beef steak and minced beef can be attributed to variations in fat content, structural integrity, and dielectric properties. The higher fat content in minced beef enhances microwave energy absorption, especially at lower frequencies like 912 MHz, which have greater penetration depth. This leads to more uniform internal heating and increased pigment reactions [29,30]. As a result, solid-state microwave cooking caused more pronounced color changes in minced beef, highlighting the importance of meat composition when evaluating cooking technologies.

### 3.5. Texture

Texture is a key quality parameter in cooked meat, and it is influenced by multiple factors such as protein denaturation, moisture loss, fat content, and muscle fiber contraction. The effects of different cooking methods on firmness and toughness in beef steak and minced beef are shown in Table 6.

In beef steak samples, both firmness and toughness increased significantly after cooking, with the greatest changes observed under microwave and solid-state microwave conditions. Firmness rose by 131% with conventional cooking, 160% with microwave cooking, and 158% with solid-state microwave cooking, compared to the raw state. Similarly, toughness increased by 54%, 72%, and 82%, respectively. These increases are likely to be due to greater moisture loss and structural tightening of muscle fibers caused by thermal denaturation and connective tissue contraction. The more intense and rapid heating in microwave-based methods may lead to faster coagulation of proteins and increased fiber rigidity, contributing to the firmer, tougher texture.

In contrast, minced beef exhibited a different pattern. Toughness decreased after cooking by 23% (conventional), 21% (microwave), and 34% (solid-state microwave), likely due to the breakdown of pre-disrupted muscle fibers and fat redistribution during heating. However, firmness still increased by 41%, 65%, and 63%, respectively. The reduction in toughness, combined with increased firmness, may reflect a softer yet more compact structure in the cooked minced samples. This may result from the fat melting and dispersing throughout the matrix, leading to less structural resistance while maintaining cohesive integrity.

The relationship between moisture content and textural parameters is also evident. As observed in the moisture analysis, higher moisture loss under microwave and solid-state microwave conditions is generally accompanied by increased firmness, especially in beef steak. This aligns with previous studies reporting that water loss contributes to tissue shrinkage and densification, which in turn increases mechanical resistance during texture analysis [31].

Overall, these results demonstrate that cooking method, meat type (whole vs. minced), and composition (e.g., fat content) significantly affect meat texture. While solid-state microwave cooking leads to the highest toughness in steak due to deeper heat penetration and more extensive denaturation, it results in a softer structure in minced beef, highlighting the complex interplay of thermal dynamics and meat microstructure.

This study was conducted under standardized experimental conditions using a fixed power level and two beef forms (steak and minced beef) to provide a controlled comparison of cooking methods. The outcomes reflect the quality attributes measured instrumentally in this work; sensory evaluation was beyond the scope of the present study and should be addressed in future research.

## 4. Conclusions

This study demonstrates that solid-state microwave cooking at 912–913 MHz offers a clear advantage in terms of reduced cooking time compared to conventional oven cooking and results in improved color development compared to traditional microwave heating. The results indicate that frequency-controlled microwave heating can influence heat transfer and quality attributes in beef steak and minced beef.

However, solid-state microwave cooking also resulted in higher weight loss and increased TBARS values, particularly in minced beef samples, indicating enhanced moisture loss and lipid oxidation under the applied conditions. These findings suggest that while solid-state microwave technology shows potential for rapid meat cooking, some quality parameters may be negatively affected if processing conditions are not carefully optimized.

From a textural and protein solubility perspective, solid-state microwave cooking produced results comparable to conventional and traditional microwave methods, with no significant differences observed among cooking techniques for several parameters. Overall, the findings highlight that solid-state microwave technology represents a promising alternative cooking method, but optimization of frequency, power, and processing conditions is necessary to minimize undesirable effects such as excessive weight loss and oxidation.

In addition, the controllability and repeatability offered by solid-state microwave systems may be advantageous for small-scale meat processing or retail applications where consistent heating is required. However, the economic feasibility and consumer sensory acceptance of this technology relative to conventional and artisanal cooking methods require further investigation.

This study was limited to fixed power levels and two meat forms (steak and minced beef). Future studies should explore a wider range of frequencies, power densities, and meat types, as well as include sensory evaluation, to better define the conditions under which solid-state microwave cooking can achieve optimal meat quality.

## Figures and Tables

**Table 1:** The cooking conditions for conventional, microwave and solid-state microwave cooking.

Cooking Parameters	Cooking Conditions		
	Conventional	Microwave	Solid-State Microwave
Cooking temperature	200 °C	NA	NA
Internal temperature	75 °C	75 °C	75 °C
Frequency	NA	2450 MHz	902–928 MHz
Power	NA	350 W	350 W
Device model	Arçelik A.Ş., AFM 340 I, İstanbul, Turkey	Arçelik A.Ş., KMF 833, İstanbul, Turkey	Custom prototype

**Table 2:** Minimum and maximum cooking times according to the application method.

	Min and Max Cooking Time (min)
Beef steak	
Conventional oven cooking	18–20
Microwave cooking	8–10
Solid-state microwave cooking	13–14
Minced Beef	
Conventional oven cooking	14–16
Microwave cooking	7–8
Solid-state microwave cooking	10–12

**Table 3:** The effect of the cooking method on weight loss, moisture, fat, and protein content in beef and minced beef.

	Weight Loss (%)	Moisture (%)	Fat (%)	Protein (%)
Beef steak				
Raw	nd	71.61 ± 4.15 ^a^	1.35 ± 0.83 ^a^	32.88 ± 6.29 ^a^
Conventional cooking	34.77 ± 4.98 ^b^	63.73 ± 1.40 ^b^	2.16 ± 1.05 ^a^	31.19 ± 1.81 ^a^
Microwave cooking	42.37 ± 4.20 ^ab^	59.28 ± 1.15 ^c^	1.94 ± 0.82 ^a^	33.68 ± 2.75 ^a^
Solid-state microwave cooking	48.12 ± 2.17 ^a^	55.70 ± 1.37 ^c^	3.04 ± 1.55 ^a^	35.34 ± 2.40 ^a^
Minced beef				
Raw	nd	57.92 ± 2.38 ^a^	14.19 ± 3.64 ^a^	26.62 ± 1.14 ^a^
Conventional cooking	24.52 ± 0.39 ^b^	59.09 ± 2.59 ^a^	11.51 ± 1.84 ^a^	25.70 ± 1.22 ^a^
Microwave cooking	26.67 ± 0.47 ^ab^	59.23 ± 4.72 ^a^	10.32 ± 1.94 ^a^	27.53 ± 4.72 ^a^
Solid-state microwave cooking	28.88 ± 2.51 ^a^	54.54 ± 0.70 ^a^	11.58 ± 1.38 ^a^	27.12 ± 1.74 ^a^

The values shown are mean ± standard deviation. Mean values indicated by different letters (^a^, ^b^, or ^c^) in the same column are statistically different (*p* ≤ 0.05). nd: not determined.

**Table 4:** The effect of the cooking method on TBARS and TSP in beef and minced beef.

	TBARS	TSP
Beef steak		
Raw	0.52 ± 0.09 ^a^	140.01 ± 9.02 ^a^
Conventional cooking	0.54 ± 0.08 ^a^	14.22 ± 5.12 ^b^
Microwave cooking	0.55 ± 0.08 ^a^	6.11 ± 2.10 ^b^
Solid state microwave cooking	0.61 ± 0.05 ^a^	9.01 ± 4.04 ^b^
Minced beef		
Raw	0.76 ± 0.16 ^c^	92.02 ± 6.12 ^a^
Conventional cooking	1.07 ± 0.05 ^b^	7.56 ± 1.71 ^b^
Microwave cooking	1.44 ± 0.07 ^a^	12.32 ± 3.63 ^b^
Solid state microwave cooking	1.56 ± 0.14 ^a^	13.12 ± 2.34 ^b^

The values shown are mean ± standard deviation. Mean values indicated by different letters (^a^, ^b^, or ^c^) in the same column are statistically different (*p* ≤ 0.05).

**Table 5:** The effect of the cooking method on color in beef and minced beef.

	L*	a*	b*	C	Hue
Beef steak					
Raw	40.80 ± 1.47 ^c^	20.25 ± 0.52 ^a^	17.43 ± 0.71 ^a^	26.71 ± 0.79 ^a^	40.71 ± 0.76 ^d^
Conventional cooking	47.61 ± 4.09 ^a^	10.01 ± 1.19 ^b^	17.65 ± 0.50 ^a^	20.34 ± 0.45 ^b^	60.58 ± 3.53 ^c^
Microwave cooking	47.37 ± 4.14 ^a^	7.26 ± 0.84 ^d^	18.14 ± 0.91 ^a^	19.57 ± 0.63 ^c^	68.11 ± 3.10 ^a^
Solid-state microwave cooking	44.12 ± 3.18 ^b^	8.47 ± 0.45 ^c^	17.91 ± 0.40 ^a^	19.76 ± 0.33 ^bc^	64.70 ± 1.56 ^b^
Minced beef					
Raw	48.38 ± 0.75 ^a^	13.60 ± 0.62 ^a^	15.40 ± 0.45 ^c^	20.55 ± 0.72 ^a^	48.81 ± 0.62 ^c^
Conventional cooking	38.31 ± 3.16 ^c^	8.48 ± 0.98 ^c^	15.51 ± 0.96 ^c^	15.80 ± 1.00 ^c^	57.63 ± 4.09 ^ab^
Microwave cooking	42.39 ± 2.83 ^b^	10.58 ± 3.25 ^b^	16.90 ± 0.25 ^a^	19.82 ± 1.48 ^a^	56.43 ± 7.23 ^b^
Solid-state microwave cooking	37.00 ± 1.43 ^c^	8.37 ± 0.63 ^c^	16.14 ± 0.43 ^b^	17.79 ± 1.25 ^b^	62.61 ± 2.09 ^a^

The values shown are mean ± standard deviation. Mean values indicated by different letters (^a^, ^b^, or ^c^) in the same column are statistically different (*p* ≤ 0.05).

**Table 6:** The effect of the cooking method on firmness and toughness in beef and minced beef.

	Firmness g	Toughness g·s
Beef steak		
Raw	615.8 ± 132.5 ^c^	3919.9 ± 685.4 ^b^
Conventional cooking	947.1 ± 166.6 ^b^	9040.9 ± 1358.1 ^a^
Microwave cooking	1061.4 ± 216.9 ^ab^	10,186.0 ± 2268.0 ^a^
Solid state microwave cooking	1118.4 ± 245.4 ^a^	10,110.3 ± 2185.3 ^a^
Minced beef		
Raw	5611.7 ± 444.0 ^a^	17,347.4 ± 7267.7 ^b^
Conventional cooking	4304.6 ± 990.5 ^b^	24,394.3 ± 7852.9 ^a^
Microwave cooking	4417.0 ± 905.2 ^b^	28,639.6 ± 5987.5 ^a^
Solid state microwave cooking	3687.2 ± 726.0 ^b^	28,318.0 ± 8558.1 ^a^

The values shown are mean ± standard deviation. Mean values indicated by different letters (^a^, ^b^, or ^c^) in the same column are statistically different (*p* ≤ 0.05).

## Data Availability

Data are available upon reasonable request from the corresponding author.
